# Clinical Trial Notifications Triggered by Artificial Intelligence–Detected Cancer Progression: A Randomized Trial

**DOI:** 10.1001/jamanetworkopen.2025.2013

**Published:** 2025-04-21

**Authors:** Tali Mazor, Karim S. Farhat, Pavel Trukhanov, James Lindsay, Matthew Galvin, Emily Mallaber, Morgan A. Paul, Michael J. Hassett, Deborah Schrag, Ethan Cerami, Kenneth L. Kehl

**Affiliations:** 1 Knowledge Systems Group, Department of Data Sciences, Dana-Farber Cancer Institute, Boston, Massachusetts; 2 Division of Population Sciences, Department of Medical Oncology, Dana-Farber Cancer Institute, Boston, Massachusetts; 3 Department of Data Sciences, Division of Population Sciences, Dana-Farber Cancer Institute, Boston, Massachusetts; 4 Department of Medicine, Memorial-Sloan Kettering Cancer Center, New York, New York

## Abstract

**Importance:**

Historically, fewer than 10% of adults with cancer have enrolled in clinical trials. Computational tools have been developed to match patients to trials, but these tools are relevant only when patients need new treatment.

**Objective:**

To evaluate whether notifying oncologists about genomically targeted clinical trials for patients with cancer progression, as detected by artificial intelligence (AI), impacts clinical trial participation.

**Design, Setting, and Participants:**

This single-center randomized trial was conducted from January 30, 2023, to June 30, 2024, at a tertiary academic cancer center. Participants were patients aged at least 18 years in a precision oncology clinical trial matching database who had solid tumors that underwent next-generation sequencing from July 2013 to December 2022, and were alive as of January 30, 2023.

**Intervention:**

Patients were randomly assigned 2:1 to the intervention or control arm. In the intervention arm, when patients had cancer progression and an elevated probability of starting new treatment based on AI applied to their imaging reports, notifications about genomically matched clinical trials were sent to their oncologists. In the control arm, no such notifications were sent.

**Main Outcomes and Measures:**

The primary outcome was enrollment in any therapeutic clinical trial. Prespecified secondary outcomes included consent to any therapeutic trial, consent and enrollment among patients ever ascertained as trial ready, the proportion of new systemic therapies that were given as part of clinical trials, and survey responses from clinicians who received notifications.

**Results:**

Of 20 707 patients randomized (57.26% female; median age at the time of sequencing, 60 years [IQR, 50-69 years]), 13 802 were randomized to the intervention arm and 6905 to the control arm. The intervention had no significant impact on the trial enrollment rate (intervention, 2.20% [95% CI, 1.97%-2.46%]; control, 2.03% [95% CI, 1.72%-2.39%]; difference, 0.18 [95% CI, −0.25 to 0.58] percentage points; *P* = .41). Similarly, there were no significant differences in trial enrollment between the intervention and control arms among the 2127 patients ever ascertained as trial ready (18.05% [95% CI, 16.15%-20.12%] vs 18.50% [95% CI, 15.78%-21.56%]; difference, −0.45 [95% CI, −4.01 to 3.02] percentage points; *P* = .80) or among the 2036 patients who ever started new systemic therapy (22.67% [95% CI, 20.51%-24.99%] vs 20.14% [95% CI, 17.33%-23.29%]; difference, 2.53 [95% CI, −1.25 to 6.21] percentage points; *P* = .19).

**Conclusions and Relevance:**

In this randomized trial, prompting academic medical oncologists with information about genomically matched therapeutic clinical trials for patients with tumor progression based on AI interpretation of imaging reports did not increase therapeutic trial enrollment. The findings suggest that future use of AI to optimize enrollment in cancer clinical trials should include tasks beyond predicting treatment change and/or populations beyond those whose tumors have undergone comprehensive genetic sequencing.

**Trial Registration:**

ClinicalTrials.gov Identifier: NCT06888089

## Key Points

**Question:**

Does sending information about genomically matched clinical trials to academic oncologists whose patients, according to an artificial intelligence (AI) model, are likely to change treatment increase trial enrollment?

**Findings:**

In this single-center randomized trial including 20 707 patients with genomically characterized solid tumors, those whose oncologists received AI-triggered notifications about genomically matched therapeutic clinical trials were no more likely to enroll in trials than were those in the control group.

**Meaning:**

The findings suggest that application of AI to optimize cancer clinical trial enrollment should include tasks beyond predicting treatment change and/or populations beyond those whose tumors have undergone comprehensive genomic sequencing at a large academic center.

## Teaser

This randomized trial examines whether notifying oncologists about genomically targeted clinical trials for eligible patients identified by artificial intelligence (AI) affects clinical trial participation.

## Introduction

Clinical trials are critical to the development of new cancer treatments. However, fewer than 10% of adults with cancer participate in treatment trials, and there are well-documented race-, sex-, and age-based disparities in trial enrollment.^1,2^ At the same time, approximately 20% of trials close prematurely, often due to low patient accrual.^3,4^ Matching patients to trials remains a complex, time consuming process that contributes to slow accrual rates and inefficiencies in the clinical research enterprise.

Several services are in development by government, academia, and industry^5^ to improve patient-trial matching.^6^ At Dana-Farber Cancer Institute (DFCI), the open-source MatchMiner tool^7^ was developed to link patients to trials using structured tumor genomic panel DNA sequencing data.^8^ MatchMiner enables oncologists to search for genomically matched trials for their patients, and it allows trial investigators to search for patients with tumors with genomic alterations targeted by their trials. Among trial enrollees, the time from genomic sequencing to consent was lower when MatchMiner was used.^7^ However, a challenge to implementation is that information about clinical trials is usually relevant only at moments when a patient needs a new treatment, usually due to disease progression. In routine practice, information about disease response and progression is recorded only in the unstructured text of a radiologist’s imaging reports or an oncologist’s clinical note. Historically, identifying patients with progressive disease has therefore required manual medical records review, which is prohibitive at scale.

Rapid advances in artificial intelligence (AI) could make clinical trial matching tools more useful in real time by extracting data on variables, such as cancer progression,^9,10,11^ from the electronic health record (EHR). Our group previously developed a model that ascertains progressive disease^9,10^ and a model that predicts whether patients will start a new systemic therapy within 30 days of an imaging report using the text of that report and prior reports for each patient.^12^ These models were piloted in MatchMiner with the goal of identifying patients potentially eligible for specific genomically targeted clinical trials at moments when they had progressive disease and were likely to change treatment.^13^ In the current study, we evaluated the impact on clinical trial participation of notifying treating oncologists about genomically matched clinical trial options when their patients had AI-ascertained progressive disease and an elevated probability of treatment change.

## Methods

### Study Design

This was a single-center, prospective randomized trial performed at DFCI among adults with solid tumors that had undergone panel DNA next-generation sequencing (NGS).^8^ The intervention consisted of emailing lists of genomically matched trials to physicians treating patients who became trial ready, defined as having AI-detected progressive disease and an elevated probability of treatment change based on imaging reports. The primary outcome was enrollment in any treatment clinical trial. This trial was registered retrospectively at the request of the journal (NCT06888089). The study protocol is given in Supplement 1. The study was approved by the Dana-Farber/Harvard Cancer Center institutional review board, with a waiver of informed consent given the minimal risk of the intervention to patients and the large cohort size. Results are reported according to the Consolidated Standards of Reporting Trials (CONSORT) reporting guideline.^14^

### Eligibility

Patients were included if they had DFCI institutional solid tumor DNA NGS (OncoPanel)^8,15^ results obtained from July 2013 to December 2022. In addition, included patients were at least 18 years of age and were alive on the randomization date of January 30, 2023.

### Study Procedures

**Figure 1.**
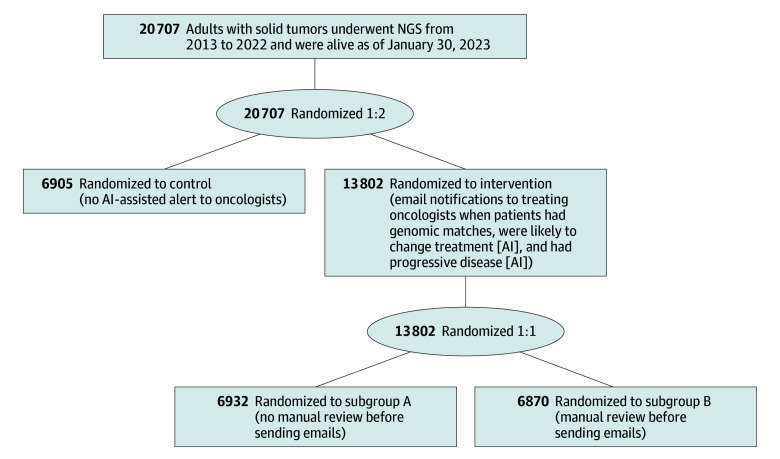
Study Schema and Patient Allocation AI indicates artificial intelligence; and NGS, next-generation sequencing.

Patients were randomized 2:1 to the intervention (AI-triggered email notifications) or control (no email notifications) arm. Randomization was not stratified given the large sample size. Randomization was performed by 1 member of the study team (M.G.), and the team member who performed final analyses (K.L.K.) did not have access to the cohort assignments until the end of follow-up. In the intervention arm, there was an additional 1:1 randomization into 2 subgroups: subgroup A had AI-triggered email notifications with no manual review before emails were sent, and subgroup B had AI-triggered email notifications with manual review (described later in this section) before emails were sent. The study schema is depicted in Figure 1. Demographic information, including race and ethnicity, was collected from the EHR. Race categories were American Indian or Alaska Native, Asian, Black or African American, White, multiracial, and unknown, and ethnicity categories were Hispanic, non-Hispanic, and unknown. Race and ethnicity data were analyzed to present balance by randomization group.

During the intervention, new imaging reports for cohort patients were pulled from the DFCI Enterprise Data Warehouse daily and processed by our AI models to extract predicted probabilities that each report described progressive disease^9^ and that the patient would start a new treatment in the next 30 days.^12^ Training and validation of these AI models were previously described.^9,12^ In brief, the model for ascertaining progressive disease was trained on 31 196 imaging reports for 2830 patients with 7 types of cancer. In a held-out test set, it yielded an area under the receiver operating characteristic curve (AUROC) of 0.95 and area under the precision recall curve (AUPRC) of 0.88 for recapitulating human annotations of progression.^9^ The model for predicting new treatment was originally trained using a dataset of 377 221 imaging reports for 20 916 patients with 434 types of cancer, yielding a held-out test AUROC of 0.77.^12^

The intervention began on January 30, 2023; the original planned end date was November 30, 2023, but after a protocol amendment, the trial enrollment rate across all arms was examined (with no analysis of outcomes by group) and the end date was extended to June 30, 2024, due to lower-than-expected overall enrollment rates. Trial consents and enrollments were recorded through 30 days after the end date. For intervention arm patients, an email containing MatchMiner^7^ genomic trial matches was sent to treating physicians each time a patient had at least 1 report demonstrating progression and at least 1 report demonstrating an elevated probability of new treatment, defined as model outputs exceeding the best *F*_1_ thresholds for those outcomes in the past week. Best *F*_1_ thresholds were defined based on validation subsets of the original data used to develop the models^9,12^ for each outcome. Treating physicians were defined as described in the eMethods in Supplement 2. Emails were sent 48 to 72 hours after imaging studies were performed given the time required for reports to be finalized and piped into the source database from which we could pull them. An example email is provided in the eMethods in Supplement 2.

Emails to physicians included genomic trial matches, surveys in which they could provide feedback on the notifications, and opt-out links with which they could request not to receive any further emails about a specific patient or not to receive any further emails at all. In the survey, physicians were asked, “We are collecting feedback on our model. Do you agree that your patient’s disease status is such that considering new treatment is reasonable?” Respondents could answer “yes,” “no,” or “already changed treatment in past week.”

For intervention arm subgroup B, a research assistant (K.S.F.) performed medical record review daily to determine whether each email would be sent. Criteria for withholding an email were predefined to include common reasons for ineligibility for most clinical trials, including uncontrolled brain metastases, poor performance status (Eastern Cooperative Oncology Group [ECOG] performance status >2), enrollment in hospice, multiple primary cancers, lack of measurable disease, AI model false positives, and already having started a new systemic therapy.

If an AI-triggered email was sent to a treating physician about a given patient, no further emails were sent for 60 days, even if the patient had more imaging reports indicating trial readiness during that time. For patients in intervention subgroup B, if an AI-triggered potential email was not sent based on the results of manual review, no additional emails were sent for 7 days if the reason for not sending the initial message was model error; otherwise, no additional emails were sent for 60 days.

### Outcomes

The primary outcome was enrollment in any treatment clinical trial. Enrollments were measured using an institutional database on July 15, 2024. Prespecified secondary outcomes included consent to any therapeutic trial, additionally capturing patients found ineligible during screening; consultation with the Center for Cancer Therapeutic Innovation (CCTI; phase 1 program) at DFCI; consent and enrollment among patients ever ascertained as trial ready; the proportion of new systemic therapies that were given on clinical trials; comparison of consent and enrollment rates between intervention subgroups A and B; survey responses from clinicians who received notifications; and rates of clinician opt-out from the notifications.

### Statistical Analysis

#### Power Calculation

Given the health system scale of our intervention, we did not prospectively recruit individual patients; instead, the cohort was defined as adults with genomically sequenced tumors alive at the beginning of the intervention. However, based on retrospective data from 11 650 patients with tumor NGS data prior to 2020 (chosen to predate the COVID-19 pandemic) who had any imaging studies during 2019 (which would trigger our notifications), we anticipated an overall trial enrollment rate of 7.5% in the control arm. Given a 2:1 split between intervention and control arms, we anticipated 80% power given 2-sided α = .05 to detect an increase in enrollment rate to 9.1% among patients with any imaging.

#### Analysis

Rates of the primary and secondary trial consent and enrollment outcomes were reported as proportions with 95% CIs. Between-group comparisons were conducted using the χ^2^ test. Two-sided *P* <  .05 was considered statistically significant. No adjustment for multiple comparisons was performed. Analysis was performed using R, version 4.3.1 (R Project for Statistical Computing) with the gmodels and crosstable packages.

## Results

### Cohort

Characteristics of the 20 707 patients included in the study (6905 patients in the control arm and 13 802 in the intervention arm) are listed in the Table. The median age at NGS was 60 years (IQR, 50-69 years); 57.26% of patients were female, 41.24% were male, and 1.50% had unknown sex. A total of 0.11% were American Indian or Alaska Native; 3.56%, Asian; 3.32%, Black or African American; 86.45%, White; 2.67%, multiracial; and 3.99%, unknown race. Overall, 3.38% were Hispanic; 95.13%, Non-Hispanic; and 1.49%, unknown ethnicity. The most common individual cancer types were non–small cell lung cancer (9.80%), colorectal cancer (8.14%), and breast cancer (7.54%).

**Table.** Patient Characteristics

Characteristic	Patients, No. (%)			
	Total (N = 20 707)	Control (n = 6905)	Intervention (n = 13 802)	
			Subgroup A (n = 6932)	Subgroup B (n = 6870)
Sex				
Female	11 857 (57.26)	3944 (57.12)	3945 (56.91)	3968 (57.76)
Male	8540 (41.24)	2864 (41.48)	2881 (41.56)	2795 (40.68)
Unknown	310 (1.50)	97 (1.40)	106 (1.53)	107 (1.56)
Cancer type				
NSCLC	2030 (9.80)	664 (9.62)	705 (10.17)	661 (9.62)
CRC	1686 (8.14)	584 (8.46)	541 (7.80)	561 (8.17)
Breast	1562 (7.54)	521 (7.55)	499 (7.20)	542 (7.89)
Glioblastoma	745 (3.60)	261 (3.78)	252 (3.64)	232 (3.38)
Pancreas	718 (3.47)	243 (3.52)	243 (3.51)	232 (3.38)
Ovarian	668 (3.23)	219 (3.17)	221 (3.19)	228 (3.32)
Melanoma	604 (2.92)	196 (2.84)	213 (3.07)	195 (2.84)
Prostate	588 (2.84)	204 (2.95)	189 (2.73)	195 (2.84)
Endometrial	483 (2.33)	171 (2.48)	133 (1.92)	179 (2.61)
Other	11 235 (54.26)	3720 (53.87)	3796 (54.76)	3719 (54.13)
Missing	388 (1.87)	122 (1.77)	140 (2.02)	126 (1.83)
Year of genomic sequencing				
2013	888 (4.29)	292 (4.23)	308 (4.44)	288 (4.19)
2014	1545 (7.46)	513 (7.43)	483 (6.97)	549 (7.99)
2015	2047 (9.89)	695 (10.07)	699 (10.08)	653 (9.51)
2016	2640 (12.75)	902 (13.06)	882 (12.72)	856 (12.46)
2017	3326 (16.06)	1073 (15.54)	1135 (16.37)	1118 (16.27)
2018	1940 (9.37)	672 (9.73)	648 (9.35)	620 (9.02)
2019	1963 (9.48)	634 (9.18)	650 (9.38)	679 (9.88)
2020	1533 (7.40)	519 (7.52)	522 (7.53)	492 (7.16)
2021	2289 (11.05)	768 (11.12)	764 (11.02)	757 (11.02)
2022	2080 (10.04)	688 (9.96)	687 (9.91)	705 (10.26)
2023^a^	30 (0.14)	12 (0.17)	13 (0.19)	5 (0.07)
Missing or unknown	426 (2.06)	137 (1.98)	141 (2.03)	148 (2.15)
Race				
American Indian or Alaskan Native	22 (0.11)	2 (0.03)	10 (0.14)	10 (0.15)
Asian	737 (3.56)	255 (3.69)	235 (3.39)	247 (3.59)
Black or African American	668 (3.32)	220 (3.19)	222 (3.20)	226 (3.29)
White	17 901 (86.45)	5951 (86.18)	6035 (87.06)	5915 (86.10)
Multiracial	552 (2.67)	190 (2.75)	167 (2.41)	195 (2.84)
Unknown	827 (3.99)	287 (4.16)	263 (3.79)	277 (4.03)
Ethnicity				
Hispanic	700 (3.38)	244 (3.53)	225 (3.25)	231 (3.36)
Non-Hispanic	19 698 (95.13)	6564 (95.06)	6602 (95.24)	6532 (95.08)
Unknown	309 (1.49)	97 (1.40)	105 (1.51)	107 (1.56)
Age at NGS, median (IQR)	60 (50-69)	60 (50-68)	60 (50-69)	60 (50-69)

Abbreviations: CRC, colorectal cancer; NGS, next-generation sequencing; NSCLC, non–small cell lung cancer.

^a^ A small number of patients with genomic sequencing ordered at the end of 2022 were reported in 2023 in our institutional database.

### Clinical Trial Consent and Enrollment Rates

**Figure 2.**
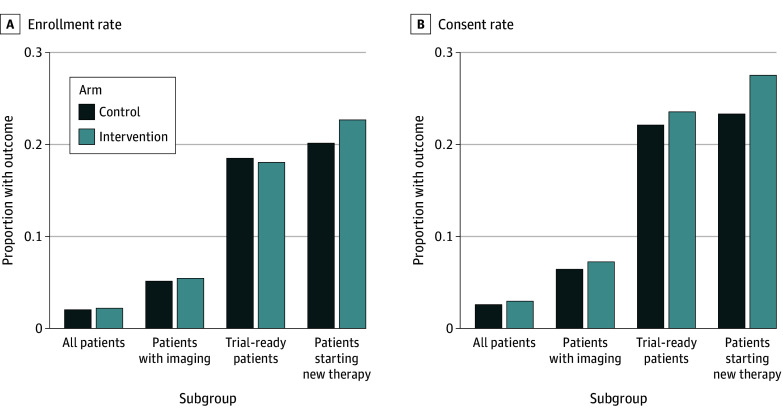
Intervention Outcomes by Subgroup Trial ready indicates ever likely to start a new treatment and found to have progressive disease by our artificial intelligence model during the study.

Outcomes are summarized in Figure 2 and eTables 1 to 4 in Supplement 2. Overall, 588 patients (2.84% of the 20 707 patients randomized) consented to a trial during the intervention, and 444 (2.14%) enrolled in a trial. There were no differences between the intervention and control arms in rates of trial consent (2.96% [95% CI, 2.69%-3.26%] vs 2.59% [95% CI, 2.24%-2.99%]; difference, 0.37 [95% CI, −0.11 to 0.83] percentage points; *P* = .13) or enrollment (2.20% [95% CI, 1.97%-2.46%] vs 2.03% [95% CI, 1.72%-2.39%]; difference, 0.18 [95% CI, −0.25 to 0.58] percentage points; *P* = .41). Similarly, there were no differences in consent (23.55% [95% CI, 21.43%-25.82%] vs 22.11% [95% CI, 19.18%-25.35%]; difference, 1.44 [95% CI, −2.39 to 5.19] percentage points; *P* = .46) or enrollment (18.05% [95% CI, 16.15%-20.12%] vs 18.50% [95% CI, 15.78%-21.56%]; difference, −0.45 [95% CI, −4.01 to 3.02] percentage points; *P* = .80) rates among the 2127 patients ascertained as trial ready at least once during the intervention.

Among the 2036 patients who started new systemic therapy during the intervention period, the rate of trial consent was nominally higher in the intervention arm than in the control arm (27.52% [95% CI, 25.19%-29.97%] vs 23.31% [95% CI, 20.32%-26.59%]; difference, 4.21 [95% CI, 0.22-8.11] percentage points; *P* = .04), but the trial enrollment rate was similar (22.67% [95% CI, 20.51%-24.99%] vs 20.14% [95% CI, 17.33%-23.29%]; difference, 2.53 [95% CI, −1.25 to 6.21] percentage points; *P* = .19). In an exploratory analysis of the 8277 patients who had any imaging performed during the intervention period, the intervention also had no significant impact on rates of trial consent (7.25% [95% CI, 6.60%-7.97%] vs 6.43% [95% CI, 5.57%-7.41%]; difference, 0.83 [95% CI, −0.34 to 1.96] percentage points; *P* = .17) or enrollment (5.45% [95% CI, 4.89%-6.08%] vs 5.14% [95% CI, 4.37%-6.04%]; difference, −0.31 [95% CI, −0.73 to 1.31] percentage points; *P* = .55) compared with the control.

There were no differences in any of these outcomes between intervention subgroup A (for whom treating oncologists received AI-triggered emails without manual review) and intervention subgroup B (for whom treating oncologists received AI-triggered emails only after manual review) (eTables 2 and 4 in Supplement 2). There were also no differences between the intervention and control arms in consent or enrollment to the genomically selected MatchMiner therapeutic trials specifically listed in the emails to treating oncologists.

### Consultations With the Early-Phase Clinical Trials Group

Among all 20 707 randomized patients, the rate of consultation with the DFCI CCTI was 0.60% (95% CI, 0.49%-0.74%) in the intervention arm compared with 0.46% (95% CI, 0.33%-0.65%) in the control arm (difference, 0.14 [95% CI, −0.08 to 0.34] percentage points; *P* = .21). Among the 2127 patients ever ascertained as trial ready, the rate was 4.81% (95% CI, 3.82%-6.04%) in the intervention arm and 3.18% (95% CI, 2.11%-4.77%) in the control arm (difference, 1.63 [95% CI, −0.18 to 3.29] percentage points; *P* = .08). Among the 2036 patients who started new systemic therapy during the study, the CCTI consultation rate was 5.22% (95% CI, 4.15%-6.54%) in the intervention arm and 3.74% (95% CI, 2.57%-5.43%) in the control arm (difference, 1.48 [95% CI, −0.45 to 3.28] percentage points; *P* = .14).

### Manual Review of Candidate Clinician Notifications

Of 1158 candidate clinician notifications manually reviewed for patients in intervention subgroup B, 269 (23.23%) were rejected (not sent). Reasons for rejection included uncontrolled brain metastases (96 [35.69%]), lack of measurable disease (76 [28.25%]), AI model false positives (46 [17.10%]), cases in which the patient had already started a new systemic therapy by the time of manual review (30 [11.15%]), ECOG performance status greater than 2 (15 [5.58%]), enrollment in hospice (11 [4.09%]), and multiple primary cancers (3 [1.12%]).

### Clinician Survey Responses

There were 2136 emails sent to 151 physicians for 1102 patients during the intervention. Among physicians, 125 (82.78%) were appointed primarily at our main academic campus and 26 (17.22%) were appointed primarily at a community-based network site (eTable 5 in Supplement 2). There were 254 clinician survey responses from 76 physicians received for 207 patients, yielding a survey response rate of 11.89% on a per-email basis and 50.33% on a per-clinician basis. Physicians agreed the patient was likely to change treatment soon in 181 responses (71.26%) or had already changed treatment in 29 responses (11.42%), and physicians disagreed in 44 responses (17.32%). On manual review of cases in which physicians disagreed with the AI prediction, common apparent reasons for disagreement included mixed response or equivocal or slow disease progression on imaging (27 [61.36%]), indication that the patient was too ill to start a new treatment (3 [6.82%]), central nervous system progression (2 [4.54%]), and clear AI model false positives (1 [2.27%]). For 5 “disagreement” responses (11.36%), there was no obvious explanation on review. No physicians opted out of notifications.

In an exploratory analysis to evaluate the association between manual review of a potential email before it was sent and clinician acceptability of the notification, survey responses were compared between intervention subgroups A and B. The proportion of survey responses indicating that the patient was likely to change treatment soon or had already done so was 83.69% (95% CI, 76.71%-88.88%) for subgroup A and 81.42% (95% CI, 73.25%-87.51%) for subgroup B (difference, −2.27 [95% CI, −11.8 to 7.10] percentage points; *P* = .63).

In another exploratory analysis to evaluate the association between physician response to the survey and patient trial enrollment, the subgroup of intervention patients whose physicians received at least 1 notification (n = 1208) was identified. In this cohort, the rate of trial consent was 28.15% (95% CI, 25.04%-31.48%) among patients whose physicians responded to at least 1 survey and 23.38% (95% CI, 19.75%-27.45%) among patients whose clinicians did not (difference, 4.7 [95% CI, −0.31 to 9.75] percentage points; *P* = .07). There was no difference in trial enrollment (21.51% [95% CI, 17.77%-23.55%] vs 19.70% [95% CI, 16.33%-23.57%]; difference, −0.81 [95% CI, −3.88 to 5.40] percentage points; *P* = .79).

### Model Performance for Predicting Treatment Change

Among 38 573 imaging reports processed during the prospective intervention, 3660 (9.49%) were followed by a new systemic therapy plan within 30 days. Our AI pipeline predicted new treatment with an AUROC of 0.85 (eFigure 1 in Supplement 2) and an AUPRC of 0.37 (eFigure 2 in Supplement 2). The best *F*_1_ threshold probability was 0.26, and the best *F*_1_ score was 0.42. At the best *F*_1_ threshold probability, the sensitivity was 52%, specificity was 90%, positive predictive value was 35%, and negative predictive value was 95% (eFigure 3 in Supplement 2). The model was well calibrated (eFigure 4 in Supplement 2).

## Discussion

In this study, previously developed AI models that ascertain progressive disease and predict initiation of new treatment based on imaging reports for patients with solid tumors were implemented at a large academic center to trigger strategically timed email prompts to treating oncologists about clinical trial options. Physicians reported that the AI-triggered notifications accurately identified patients likely to change treatment, which was confirmed quantitatively via prospective evaluation of the performance of our underlying AI model. As expected, since many factors influence initiation of new treatment besides imaging findings, the model did not perfectly predict treatment changes. Nevertheless, it was able to identify the patients most likely to change treatment. Still, the notifications did not increase clinical trial consent or enrollment rates.

These findings beg the question of what is required to translate well-performing AI models into clinical decision support that improves care delivery and/or outcomes. Most randomized trials of clinical AI have focused on computer vision tasks.^16^ Few prospective randomized studies of AI-triggered nudges to clinicians in oncology have been conducted. One study^17^ demonstrated that contacting clinicians whose patients had a high probability of 6-month mortality could increase rates of serious illness conversations and decrease end-of-life systemic therapy, although there was no effect on hospice enrollment or length of stay, inpatient death, or intensive care unit use at the end of life.

### Strengths and Limitations

Strengths of this study include a large cohort, application of validated AI models that performed well prospectively during the intervention, and multidimensional assessment of the acceptability and utility of the notifications. This study also has limitations. Overall, the impact of our intervention may have been limited by its focus on identifying patients with progressive disease to target information about potential genomically targeted clinical trials, which addresses only 1 of many barriers to trial participation. In a previous pilot study^13^ focused on identifying patients for specific early-phase genomically focused clinical trials, our group found that multiple factors contributed to ineligibility for trials on manual review. These included cases in which patients had already changed treatment or already decided to continue treatment, the trial had no available slots, or the patient was not eligible based on clinical criteria other than genomics.

Additional specific limitations included a lower clinical trial enrollment rate than we had anticipated. This was likely due both to residual effects of the COVID-19 pandemic early in our intervention and to a design that did not progressively incorporate patients with new tumor sequencing results, who might have had more active disease and required more treatment changes. The randomized cohort also included any patients with tumor NGS results without regard to disease status since we could not know in advance which patients would have active disease requiring treatment during the intervention. For this reason, however, we analyzed secondary outcomes focused on patients with active disease during the intervention, including those who had any imaging, had imaging and were deemed likely to change treatment by our AI model, and started any new systemic therapy.

Another limitation was the focus on genomically targeted trials at a single academic center, which has thus far been the scope of our MatchMiner tool. In this context, many oncologists were likely already familiar with trial options in their own subspecialty area of practice, although some may not have known about early-phase trials in our dedicated phase 1 trial center. Providing trial information to oncologists in a community setting, especially with matching based on variables beyond genomics and progressive disease, might have a larger impact. This could further expand utility for patients who need a new treatment in the absence of progressive disease, such as those with de novo advanced disease, who need neoadjuvant or adjuvant therapy, or who discontinued prior treatment due to adverse events. Oncologist engagement with the notifications was likely variable. Approximately half of oncologists who received notifications provided feedback in our survey at least once (50.33%), but for busy physicians, it may be challenging for one more source of notifications to have a significant impact on clinical workflows.

The delay of 48 to 72 hours between generation of an imaging report and generation of a notification, which was due to the mechanisms of clinical data flow from our EHR to a repository on which notifications can be computed, may also have diminished notification impact for patients who started a new treatment as soon as progressive disease was identified. Still, the routine workflow for a new standard-of-care therapy at our center involves allowing 7 days to obtain insurance authorization; thus, this should not have been prohibitive. The email means of notification also allowed asynchronous delivery of clinical trial information outside clinical encounters, potentially facilitating planning for the next visit.

Our primary outcome was enrollment to any therapeutic trial, even if it was not a specific trial highlighted by MatchMiner, since in our group’s previous pilot study,^13^ we learned that patients referred to our early drug development center to consider one trial often eventually enrolled in a different trial. This could have diluted the impact of our intervention. Still, in an exploratory analysis, we saw no difference in enrollment to genomically targeted trials specifically in the MatchMiner database.

## Conclusions

In this randomized trial, proactive oncologist notification about genomically matched clinical trials when patients had progressive disease ascertained using AI models did not increase therapeutic clinical trial enrollment rates at a large academic cancer center. The findings suggest that using AI to optimize enrollment in cancer clinical trials should include factors beyond predicting treatment change and/or populations beyond those whose tumors have undergone comprehensive NGS.

## Notes

Supplement 1.
Study Protocol

Supplement 2. **eTable 1.** Clinical Trial Enrollments by Intervention Group **eTable 2.** Clinical Trial Enrollments by Intervention Subgroup **eTable 3.** Clinical Trial Consents by Intervention Group **eTable 4.** Clinical Trial Consents by Intervention Subgroup **eTable 5.** Characteristics of Physicians Who Received Notifications **eFigure 1.** Area Under Receiver Operating Characteristic Curve of AI Model for Predicting New Treatment **eFigure 2.** Area Under Precision Recall Curve for Predicting New Treatment **eFigure 3.** Confusion Matrix for Predicting New Treatment at the Best *F*_1_ Threshold **eFigure 4.** Distribution of New Treatment Predictions and Calibration Curve for Predicting New Treatment
eMethods.

Supplement 3.
Data Sharing Statement
