# Supplementary material for: Clinical Trial Notifications Triggered by Artificial Intelligence–Detected Cancer Progression: A Randomized Trial
Source: JAMA Netw Open. 2025 Apr 21;8(4):e252013. doi: 10.1001/jamanetworkopen.2025.2013 (PMC12013351; doi:10.1001/jamanetworkopen.2025.2013)
Supplement: Supplement 1. — Study Protocol [file jamanetwopen-e252013-s001.pdf]

PROTOCOL TITLE:

Deep clinical trajectory modeling to optimize accrual to cancer clinical trials

PRINCIPAL INVESTIGATOR:

Kenneth L. Kehl, MD, MPH
Population Sciences Department
617-632-4550
Kenneth_Kehl@dfci.harvard.edu

VERSION NUMBER:

Version 3

DATE:

7/9/23

1.0 Objectives 3
2.0 Background 3
3.0 Inclusion and Exclusion Criteria 3
4.0 Study-Wide Number of Subjects 4
5.0 Study-Wide Recruitment Methods 4
6.0 Multi-Site Research 5
7.0 Study Timelines 5
8.0 Study Endpoints 5
9.0 Procedures Involved 5
10.0 Data and Specimen Banking 5
11.0 Data Management and Confidentiality 9
12.0 Provisions to Monitor the Data to Ensure the Safety of Subjects 9
13.0 Withdrawal of Subjects 9
14.0 Risks to Subjects 9
15.0 Potential Benefits to Subjects 9
16.0 Vulnerable Populations 9
17.0 Community-Based Participatory Research 9
18.0 Sharing of Results with Subjects 9
19.0 Setting 9
20.0 Resources Available 10
21.0 Prior Approvals 10
22.0 Recruitment Methods 10
23.0 Local Number of Subjects 10
24.0 Provisions to Protect the Privacy Interests of Subjects 10
25.0 Compensation for Research-Related Injury 10
26.0 Economic Burden to Subjects 10
27.0 Consent Process 11
28.0 Process to Document Consent in Writing 11
29.0 Drugs or Devices 11
29.0 References 11

1.0 Objectives

The goals of this project are to develop deep learning models that can represent patients' clinical trajectories based on text data within electronic health records, and to evaluate the feasibility of incorporating these models into the existing MatchMiner institutional clinical trial matching tool to optimize accrual rates to biomarker-selected therapeutic clinical trials. The study will include an algorithm development and a model implementation component. The algorithm development component will be a mixed retrospective and prospective analysis of medical records data for patients who have undergone OncoPanel genomic testing from 2013-2022. Structured and unstructured data for these patients will be requested using the OncDRS infrastructure, with monthly refreshes requested to include continuously up-to-date records.

The model implementation component will apply these models as new medical records are generated, in order to evaluate the impact on clinical trial accrual of providing information from the Dana-Farber MatchMiner tool to providers and principal investigators when patients appear 'ready' for a clinical trial based on algorithmic predictions. MatchMiner is an established clinical operations tool at DFCI that links OncoPanel data to basic clinical information and clinical trial eligibility criteria to suggest biomarker-selected therapeutic trials for patients.

Our project will itself involve no direct patient contact, no interventions directly upon patients, and no assessment of interventions on health-related patient outcomes; it is a cancer care delivery/clinical operations research study. This proposal therefore does not comprise a therapeutic clinical trial in its own right. The outcomes of our prospective component will be evaluation in the Center for Cancer Therapeutic Innovation; rates of consent to any therapeutic clinical trial; and rates of enrollment in any therapeutic clinical trial during the implementation study. Clinicians will continue to decide whether to approach any patient flagged as a potential therapeutic trial candidate, and informed consent would be obtained for those therapeutic trials in the usual manner. Algorithm development to date has proceeded under protocol 16-360. The current protocol proposes to apply these models to optimize delivery of information from the MatchMiner tool.

The overall study objectives are to:

1. Develop and validate a clinically relevant, dynamic, pre-trained cancer trajectory model by applying deep learning to integrated structured and unstructured EHR data.

2. Apply transfer learning to a pre-trained cancer trajectory model to match patients to clinical trials using EHR data and clinical trial protocols.

3. Pilot the incorporation of cancer trajectory modeling into clinical trial accrual efforts for patients whose tumors have undergone next-generation sequencing.

2.0 Background

The U.S. healthcare system has rapidly incorporated electronic health records (EHRs) into routine clinical practice over the last ten years.[1] In oncology, the large volume of data contained within EHRs could constitute a source of 'real-world evidence'[2] to drive research and optimize care delivery. However, most of this information remains untapped. Key oncologic variables, such as

disease histology and stage, often exist in the EHR only in unstructured form. Even when structured data are available, they may capture cancer status only at one point at time, without capturing dynamic shifts in phenotype occurring across the disease trajectory. Without this information, identifying cohorts of patients eligible for research, quality improvement efforts, and clinical trials requires resource-intensive manual medical records review, which may be prohibitive at scale.

Clinical trial enrollment rates among adults with cancer have historically been under 5%.[3,4] Despite the large population of patients who do not enroll in trials, studies often struggle to reach their accrual goals.[5,6] A variety of services are in development by government, academia, and industry[7] to improve matching between patients and available trials,[8] but no such frameworks both incorporate unstructured EHR data and are readily available to the public. At Dana-Farber Cancer Institute (DFCI), the MatchMiner tool[9] has been developed to link patients to clinical trials using structured genomic data.[10] However, MatchMiner does not yet contain a mechanism for specifically identifying clinically eligible patients when they most need a new treatment.

Rapid innovation in deep learning techniques may provide novel solutions to these challenges. In recent work, we have found that deep learning classifiers can accurately extract key oncologic outcomes from radiology report text.[11] Deep learning additionally facilitates transfer learning,[12] in which models pre-trained for one purpose can be applied to related problems, using less labeled data than might otherwise be required. It can further readily be applied to classification and prediction problems using sequences of input and multiple types of data.[13] Pre-trained deep learning methods that represent both static (e.g., histology) and dynamic (e.g., cancer progression) oncologic features using a complex array of structured and unstructured EHR data could constitute a transformative technology in support of cohort definition and prediction tasks.

The goals of this proposal are to develop scalable deep learning methods for capturing a patient’s cancer status and trajectory using EHR data, and to apply these techniques to build an open framework for improving accrual to clinical trials.

3.0 Inclusion and Exclusion Criteria

The cohort will consist of adults with any type of cancer whose tumors underwent OncoPanel genomic sequencing from 2013-2022. Children will not be included, due to incomplete capture of the EHR data necessary to train machine learning models, given the high proportion of care delivered at Children’s Hospital Boston for children with cancer. OncDRS will be utilized to identify all patients and assess for eligibility for this study.

4.0 Study-Wide Number of Subjects

This is a medical record data analysis and health system implementation project using data files from Dana-Farber Cancer Institute (DFCI) and satellite sites. The study type consists of <u>combined</u> *retrospective* medical record review, *prospective* OncDRS data refreshes, and prospective implementation.

- Retrospective Review Component: The study team ran an aggregate query request in OncDRS as of April 2022 to see how many patients had undergone OncoPanel sequencing by that date. This query resulted in approximately 51,417 patients.

- Prospective Review Component: We will refresh the OncDRS data query/request once a year to capture new cases.

For this project, up to 60,000 records could be pulled for algorithm development and MatchMiner enhancement. We will not exceed 60,000 record reviews without prior IRB approval.

5.0 Study-Wide Recruitment Methods

Not applicable – The cohort for this project is defined as those patients whose tumors have undergone next-generation sequencing either on a research basis under protocols 11-104 or 17-000, or on a clinical basis via Clinical Oncopanel. The current project therefore does not involve direct patient recruitment.

6.0 Multi-Site Research

Not applicable – This is NOT a multi-site research project.

7.0 Study Timelines

Initial algorithm development is complete, and algorithms are being refined based on our prospective pilot. We will continue to pilot delivery of tailored MatchMiner information to disease centers including the Center for Cancer Therapeutic Innovation, the Breast Oncology Center, and the Thoracic Oncology program through 2025. In the second half of 2022, a retrospective data analysis and medical record review will be undertaken to evaluate the sensitivity, specificity, positive predictive value, and negative predictive value of our NLP models for predicting clinical trial consent and enrollment, and to evaluate reasons for non-enrollment in trials. During 2023, we will undertake the prospective component of our intervention, which will evaluate the impact of different strategies for delivering MatchMiner information on clinical trial evaluation, consent, and enrollment rates.

8.0 Study Endpoints

For algorithm development using EHR data, outcomes for model training will include disease progression, defined using a structured manual annotation framework for model training (protocol 16-360); initiation of new systemic therapies; evaluation in the Center for Cancer Therapeutic Innovation; consents to therapeutic clinical trials; enrollments in therapeutic trials; and overall survival.

The primary endpoint that will be assessed by the interventional component of this study is whether each patient with NGS (OncoPanel) data enrolls in any therapeutic clinical trial within one month following the end of the intervention period. Key secondary endpoints will include consultations with the Center for Cancer Therapeutic Innovation and rates of consent to any therapeutic clinical trial during that period.

9.0 Procedures Involved

The algorithm development component of this study has included a medical record analysis project using Dana-Farber Cancer Institute data files and involves no medical procedures or treatments per se. This work is currently underway per protocol 16-360, and it involves analysis of records dating from 1997-2022 for OncoPanel patients for whom genomic testing was ordered

from 2013-2022. These records are used to train machine learning/natural language processing models to predict changes in treatment, prognosis, progression of disease, and sites of metastatic disease using retrospective imaging reports. These models can then be applied prospectively to identify patients who may be appropriate candidates for clinical trials at specific moments in time due to a high propensity to change treatment in the near future.

For the intervention component, there will be two phases.

The first phase of the intervention component will consist of a pilot phase in which our AI algorithms for predicting treatment changes and prognosis are incorporated into the MatchMiner "patients for clinical trials" workflow. Specifically, filters already generated by the MatchMiner team to identify patients who may be candidates for specific clinical trials based on NGS data will be cross-referenced with predictions of new treatment and prognosis that are generated each time a patient has an imaging report generated for cancer reassessment. This combined information will be used to sort the list of filter patients by propensity to change treatment as well as timing of most recent progression and brain metastasis. These sorted MatchMiner lists will then be delivered as spreadsheets to collaborating investigators in the Center for Cancer Therapeutic Innovation, the Breast Oncology Center, and the Thoracic Oncology Program, and qualitative feedback will be solicited regarding steps to improve our modeling and/or delivery of information. Feedback will be collected via a structured SurveyMonkey survey every six months and informally via meetings with these disease centers. These collaborators are co-investigators on our protocol, not research subjects. Survey questions will ask respondents to provide their disease center; role (MD, RN NP/PA, other); estimates of how often they review trial readiness spreadsheets; estimates of the extent to which the readiness spreadsheets have been incorporated into disease center team workflows; estimates of the subjective accuracy of trial readiness predictions; estimates of whether patients have consented to trials or enrolled on trials specifically because of these predictions; feature requests; any ethical concerns; and whether readiness spreadsheets are being used in disease centers for any purposes other than identifying patients for clinical trials (eg maintaining disease center databases, identifying patients who may need palliative care services). CCTI will also review each 'ready' patient in our spreadsheets to indicate whether a patient was indeed discussed with the treating clinician for possible referral to CCTI; and if not, why not.

The second phase of the intervention component will consist of a pragmatic, prospective, randomized health care system process implementation study. This is not a clinical trial by the NIH definition, since the outcomes assessed will be operational rather than patient-level behavioral or health-related outcomes.

The intervention procedure for this second phase will be:

- On the study go-live date, which is currently anticipated to be January 2, 2023 but may be adjusted if needed based on technical factors identified during the pilot phase, we will identify all patients with OncoPanel results (who therefore have data accessible through MatchMiner already), who are still alive at that date, whose tumor NGS included any OncoTree code other than leukemia, lymphoma, myeloma, or MDS codes; and who are age 18 or over on that date.

- Such patients will be randomly divided into three groups; randomization will be performed at the patient level. For Group 1, MatchMiner will operate as it has previously; clinicians can use the software to identify potential trials for their patients based on NGS data, and clinical trial investigators can use it to identify potential patients for their trials. For Group 2, MatchMiner will continue to operate as before, but additionally, email notifications containing MatchMiner information will be automatically sent to treating oncologists each time a patient is clinical trial "ready" (has a predicted probability of treatment change that falls above the best F1 cutoff threshold for this prediction as evaluated in the retrospective validation set, also has MatchMiner trial matches for trials on which the patient has not previously enrolled, and has not triggered a recent "readiness" email to the treating clinician). For Group 3, MatchMiner will still continue to operate as before, with NGS-based matches available to all treating oncologists and trial investigators, and additionally, lists of "ready" patients will be generated based on AI model results as in Group 2; however, these lists will be manually reviewed by the study team and cross-referenced with manual medical record review before the MatchMiner notifications are emailed to treating oncologists. Manual review criteria for proceeding with each email will include evaluation of whether patients have already been referred to hospice or are being treated with comfort measures only at the time of the last imaging report; whether patients have documented performance status of 3-4; and whether patients are obviously ineligible for all trials provided in the MatchMiner report as evaluable in the medical record based on trial-specific eligibility criteria. There will be no direct contact with patients in this study.

- We have collected preliminary data to inform the randomization and analysis strategy. As of February 2022, there were 46,097 patients with tumor NGS (OncoPanel) data.. However, this includes both living and deceased patients, patients with hematologic malignancies who will not be included in this study, and patients not actively following at DFCI. We therefore examined our AI algorithm development data from 2019 (to exclude COVID-related variability in trial accrual). We estimate that there were 11,650 patients who had NGS data and who had any imaging studies from January 1, 2019 through October 31, 2019. Of these patients, approximately 890 (7.6%) enrolled in a therapeutic clinical trial between January 1, 2019 and November 30, 2019. If we perform 1:1:1 randomization for all living patients with NGS and given growth in the PROFILE cohort since 2019, we will therefore have approximately 3800 patients in each group who have imaging studies during our ten-month intervention period. Stratified randomization will not be performed, due to the large sample size and heterogeneity in treating disease centers, treating clinicians, cancer types. Clinicians will be given the opportunity to opt out of ongoing emails from the study in general, or for individual patients, at any time. Patients whose clinicians opt out of notifications will remain in their assigned group for analysis; that is, the primary analysis will follow an intention to treat approach.

The primary study outcome will be whether patients enroll on any DFCI therapeutic clinical trial of an anti-cancer systemic therapy during the intervention follow-up period. This period will be defined as follows. Beginning in December 2023, total trial

enrollment events across all three study arms will be quantified every two months, without comparing enrollment among arms; the goal of this procedure is to ensure adequate statistical power to compare among arms. Once total trial enrollment reaches 1000 patients, or the follow-up period reaches July 1, 2024, the study will be discontinued and outcomes by arm assessed. Trial enrollment will be ascertained using structured institutional data via an existing data feed from the Dana-Farber Enterprise Data Warehouse to the MatchMiner team. In our primary analysis, we will pool the two AI-assisted intervention arms and compare trial enrollment rates in the AI-assisted group (N~7600 total) to the standard process arm (N~3500) using a simple two-sample Z test of proportions. If the standard process arm enrollment rate remains ~ 7.5%, we would have 80% power given two-sided alpha=0.05 to detect an increase in enrollment rate to 9.1%. This would represent an operationally meaningful 21% relative increase in enrollment rates. We will take this approach rather than a time-to-event analysis, since traditional right censoring, in which patients are lost to follow-up but undetected events might have occurred after they were no longer followed, is not relevant for this outcome; we will have complete data on the outcome from DFCI (unascertained enrollment in a therapeutic trial would not be possible).

Comparison of the two intervention arms (Groups 2 and 3) to each other and to Group 1 will be a pre-specified secondary analysis. Additional pre-specified secondary outcomes will include whether patients have any consultations in the Center for Cancer Therapeutic Innovation and whether they consent to any therapeutic trial. Further pre-specified secondary outcomes will include trial consent and enrollment rates among patients who at any point during follow-up were ascertained as likely to change treatment in the next 30 days as defined by our algorithm; the proportion of new systemic therapy regimens that were clinical trials; and clinician opt-out rates. Of note, these are administrative/process outcomes, not clinical outcomes, such that our intervention does not itself constitute a clinical trial by the NIH definition. These endpoints will also be analyzed using the MatchMiner EDW data feed. Furthermore, the intervention consists of comparing ways to deliver information from the MatchMiner tool that is already accessible to treating clinicians, such that there is minimal risk to patients and clinicians.

The pilot phase work will be downsized when the interventional phase begins to minimize impact of the pilot on evaluation of the intervention. Specifically, the Breast Oncology Center and Thoracic Oncology Program components of the pilot will be discontinued, and the trials involved in the Center for Cancer Therapeutic Innovation (CCTI) pilot will be restricted to a subset of trials for rare patient populations for which CCTI collaborators determine there is a strong need to identify as many potential patients as possible. This list of trials will be determined at the time of intervention go-live, since it is challenging to predict trial portfolios far in advance.

This will be an interventional research study, but it will not itself constitute a clinical trial by the NIH definition, since the outcomes being measured (whether patients consent to or enroll on therapeutic clinical trials) are not themselves health-related outcomes for the patient.

10.0 Data and Specimen Banking

Not applicable

11.0 Data Management and Confidentiality

This is a medical record analysis and health system process implementation study; it does not involve direct patient contact, and the research could not be done if contacting all ~60,000 patients for algorithm development and implementation were required. Furthermore, the implementation component of our study in MatchMiner addresses operational outcomes rather than biomedical patient-level outcomes; it similarly involves no patient contact. The medical risk to patients will be minimal, since their clinicians will continue to make all relevant standard-of-care treatment or therapeutic clinical trial recommendations. As such, a waiver of informed consent will be requested. The principal theoretical risk in the medical records review component of the study is loss of confidentiality. All structured and unstructured data obtained through OncDRS for this study will be stored only in secure password-protected DFCI-approved storage locations, limited to Google Cloud Storage (per a Business Associate Agreement between DFCI Clinical Informatics and Google to provide HIPAA-compliant data storage), Partners DropBox, DFCI Redcap, or servers housed in the DFCI Department of Data Sciences. Only study investigators will have access to the data.

12.0 Provisions to Monitor the Data to Ensure the Safety of Subjects

Not applicable

13.0 Withdrawal of Subjects

Not applicable

14.0 Risks to Subjects

As above, this will be a medical record algorithm development and operational process research study without patient contact; as such a waiver of informed consent will be requested. The principal theoretical risk in any medical records review study is loss of confidentiality, which will be addressed using the measures in Section 11 above.

15.0 Potential Benefits to Subjects

This project does not include a direct benefit for subjects included in the analysis.

16.0 Vulnerable Populations

Not applicable

17.0 Community-Based Participatory Research

Not applicable

18.0 Sharing of Results with Subjects

Not applicable

19.0 Setting

This is a medical record analysis project using Dana-Farber Cancer Institute data files and an operational process study to be conducted at Dana-Farber Cancer Institute.

20.0 Resources Available

The Population Sciences Division maintains its own server infrastructure and systems administration staff that provide data storage, data backup, and data security in support of large data analysis projects. The servers are configured as a virtual server pool with virtual server hosts connected to a centralized Storage Area Network (SAN) device. Server virtualization increases the efficiency and flexibility of the server pool while minimizing downtime and cost. The server pool currently has 20 processor cores and a data storage capacity of 9 terabytes. This server infrastructure has a dedicated Systems Administrator to optimize performance, maintain security patches, perform backups, and execute other related tasks. Researchers in the Division have access to additional resources through the Research Computing group. Research Computing provides a variety of services including file server space, backup services, website hosting, and support of some workstation computers.

21.0 Prior Approvals

Not applicable

22.0 Recruitment Methods

Not applicable

23.0 Local Number of Subjects

Not applicable

24.0 Provisions to Protect the Privacy Interests of Subjects

There will be no direct patient contact in this study. The algorithm development study includes a Combined *retrospective* review with *prospective* OncDRS data refreshes. The OncDRS refreshes will allow us to update the cohort over time. Therefore, we recognize that not every piece of data for this project is on the shelf as of today. However, every piece of data will ONLY be utilized via medical record review and OncDRS refreshes. There will be NO direct patient interaction during this project. The principal theoretical risk in any medical records review study is loss of confidentiality. The data obtained for this study will be stored in DFCI-approved server locations, accessible only to study staff, as described above. In the implementation component of the study, filtered MatchMiner results are being delivered to collaborators in disease centers who are also investigators on this protocol; prospective notifications to treating oncologists will be delivered using the MGB email system as is other patient-related information.

25.0 Compensation for Research-Related Injury

Not applicable

26.0 Economic Burden to Subjects

Not applicable

27.0 Consent Process

This will be a medical record cohort study and health system operational study without patient contact; as such a waiver of informed consent will be requested from the DF/HCC IRB. We are requesting a waiver of consent because this is a minimal risk study that involves only retrospective data review and prospective implementation of models to improve the operation of the MatchMiner tool for prioritizing actual therapeutic clinical trials for patients. There will never be any direct interaction with subjects in our study. Also of note, due to the large anticipated cohort for this study (n=60,000 subjects), it is not feasible to consent all participants. Additionally, precautions will be taken to ensure that data is secure, including locking physical materials and storing electronic materials on a secure server.

28.0 Process to Document Consent in Writing

Not applicable

29.0 Drugs or Devices

Not applicable

30.0 References

1. Washington V, DeSalvo K, Mostashari F, Blumenthal D. The HITECH Era and the Path Forward. *N Engl J Med*. 2017;377:904-906.
2. Sherman RE, Anderson SA, Dal Pan GJ, et al. Real-World Evidence — What Is It and What Can It Tell Us? *N Engl J Med*. 2016;375:2293-2297.
3. Kehl KL, Arora NK, Schrag D, et al. Discussions About Clinical Trials Among Patients With Newly Diagnosed Lung and Colorectal Cancer. *J Natl Cancer Inst*. 2014;106:1-9.
4. Fouad MN, Lee JY, Catalano PJ, et al. Enrollment of patients with lung and colorectal cancers onto clinical trials. *J Oncol Pract*. 2013;9:e40-7.
5. Nass SJ, Moses HL, Mendelsohn J. *A National Cancer Clinical Trials System for the 21st Century: Reinvigorating the NCI Cooperative Group Program.* Committee on Cancer Clinical Trials and the NCI Cooperative Group Program; Institute of Medicine, National Academies Press; 2010.
6. Schroen AT, Petroni GR, Wang H, et al. Achieving sufficient accrual to address the primary endpoint in phase III clinical trials from U.S. Cooperative Oncology Groups. *Clin Cancer Res*. 2012;18:256-262.
7. IBM Watson for Clinical Trial Matching. https://www.ibm.com/us-en/marketplace/watson-for-clinical-trial-matching/details. Accessed January 27, 2019.
8. American Cancer Society. An Overview of Cancer Clinical Trial Matching Services. www.acscan.org. Accessed February 1, 2019.
9. Lindsay J, Fitz CDV, Zwiesler Z, et al. MatchMiner: An open source computational platform for real-time matching of cancer patients to precision medicine clinical trials using genomic and clinical criteria. *bioRxiv*. 2017.
10. Sholl LM, Do K, Shivdasani P, et al. Institutional implementation of clinical tumor profiling on an unselected cancer population. *JCI Insight*. 2016;1.
11. Kehl KL, Elmarakeby H, Nishino M, et al. Assessment of Deep Natural Language Processing in Ascertaining Oncologic Outcomes From Radiology Reports. *JAMA Oncol*. 2019;02215:1-8.

12. Pan SJ, Yang Q. A Survey on Transfer Learning. *IEEE Trans Knowl Data Eng*. 2010;22:1345-1359.
13. Wainberg M, Merico D, Delong A, Frey BJ. Deep learning in biomedicine. *Nat Biotechnol*. 2018;36:829-838.
