# Supplementary material for: Clinical Trial Notifications Triggered by Artificial Intelligence–Detected Cancer Progression: A Randomized Trial
Source: JAMA Netw Open. 2025 Apr 21;8(4):e252013. doi: 10.1001/jamanetworkopen.2025.2013 (PMC12013351; doi:10.1001/jamanetworkopen.2025.2013)
Supplement: Supplement 2. — eTable 1. Clinical Trial Enrollments by Intervention Group eTable 2. Clinical Trial Enrollments by Intervention Subgroup eTable 3. Clinical Trial Consents by Intervention Group eTable 4. Clinical Trial Consents by Intervention Subgroup eTable 5. Characteristics of Physicians Who Received Notifications eFigure 1. Area Under Receiver Operating Characteristic Curve of AI Model for Predicting New Treatment eFigure 2. Area Under Precision Recall Curve for Predicting New Treatment eFigure 3. Confusion Matrix for Predicting New Treatment at the Best F1 Threshold eFigure 4. Distribution of New Treatment Predictions and Calibration Curve for Predicting New Treatment eMethods. [file jamanetwopen-e252013-s002.pdf]

Supplementary Online Content

Mazor T, Farhat KS, Trukhanov P, et al. Clinical trial notifications triggered by artificial intelligence–detected cancer progression: a randomized trial. *JAMA Netw Open*. 2025;8(4):e252013. doi:10.1001/jamanetworkopen.2025.2013

eTable 1. Clinical Trial Enrollments by Intervention Group
eTable 2. Clinical Trial Enrollments by Intervention Subgroup
eTable 3. Clinical Trial Consents by Intervention Group
eTable 4. Clinical Trial Consents by Intervention Subgroup
eTable 5. Characteristics of Physicians Who Received Notifications
eFigure 1. Area Under Receiver Operating Characteristic Curve of AI Model for Predicting New Treatment
eFigure 2. Area Under Precision Recall Curve for Predicting New Treatment
eFigure 3. Confusion Matrix for Predicting New Treatment at the Best F_1 Threshold
eFigure 4. Distribution of New Treatment Predictions and Calibration Curve for Predicting New Treatment
eMethods.

This supplementary material has been provided by the authors to give readers additional information about their work.

© 2025 Mazor T et al. *JAMA Network Open*.

eTable 1: Clinical trial enrollments by intervention group

Denominator	Control group trial enrollment rate (%, 95% CI)	Intervention group trial enrollment rate (%, 95% CI)	Absolute enrollment rate difference (%, 95% CI)	P
Randomized patients (n=20707)	2.03 (1.72-2.39)	2.20 (1.97-2.46)	0.18 (-0.25 to 0.58)	0.41
Randomized patients who had imaging studies (n=8277)	5.14 (4.37-6.04)	5.45 (4.89-6.08)	0.31 (-0.73 to 1.31)	0.55
Randomized patients who had imaging studies and were ever "ready" per AI model (n=2127)	18.50 (15.78-21.56)	18.05 (16.15-20.12)	-0.45 (-4.01 to 3.02)	0.80
Randomized patients who started any new systemic therapy (n=2036)	20.14 (17.33-23.29)	22.67 (20.5-24.99)	2.53 (-1.25 to 6.21)	0.19

© 2025 Mazor T et al. *JAMA Network Open*.

eTable 2: Clinical trial enrollments by intervention subgroup

	Control group	Intervention group	
Denominator	Enrollment rate (%, 95% CI)	Subgroup A enrollment rate (%, 95% CI)	Subgroup B enrollment rate (%, 95% CI)
Randomized patients (n=20707)	2.03 (1.72-2.39)	2.16 (1.85-2.53)	2.24 (1.92-2.62)
Randomized patients who had imaging studies (n=8277)	5.14 (4.37-6.04)	5.41 (4.63-6.32)	5.49 (4.71-6.40)
Randomized patients who had imaging studies and were ever "ready" per AI model (n=2127)	18.50 (15.78-21.56)	17.73 (15.09-20.72)	18.36 (15.72-21.33)
Randomized patients who started any new systemic therapy (n=2036)	20.14 (17.33-23.29)	22.49 (19.48-25.81)	22.85 (19.84-26.17)

Legend to eTable 2: Control group, control group with standard MatchMiner workflows; Subgroup A, intervention subgroup in which emails were automatically sent to treating oncologists when patients had genomic matches to a trial and were predicted likely to change treatment by our AI model; Subgroup B, intervention subgroup in which emails were sent to treating oncologists when patients had genomic matches to a trial, were predicted likely to change treatment, and did not meet exclusions on manual review. Manual review exclusions included uncontrolled brain metastases; poor performance status (ECOG performance status > 2); enrollment on hospice; multiple primary cancers; lack of measurable disease; AI model false positives; and already having received a new systemic therapy.

© 2025 Mazor T et al. *JAMA Network Open*.

eTable 3: Clinical trial consents by intervention group

Denominator	Control group trial consent rate (%, 95% CI)	Intervention group trial consent rate (%, 95% CI)	Absolute consent rate difference (%, 95% CI)	P
Randomized patients (n=20707)	2.59 (2.24-2.99)	2.96 (2.69-3.26)	0.37 (-0.11 to 0.83)	0.13
Randomized patients who had imaging studies (n=8277)	6.43 (5.57-7.41)	7.25 (6.60-7.97)	0.83 (-0.34 to 1.96)	0.17
Randomized patients who had imaging studies and were ever "ready" per AI model (n=2127)	22.11 (19.18-25.35)	23.55 (21.43-25.82)	1.44 (-2.39 to 5.19)	0.46
Randomized patients who started any new systemic therapy (n=2036)	23.31 (20.32-26.59)	27.52 (25.19-29.97)	4.21 (0.22 to 8.11)	0.04

© 2025 Mazor T et al. *JAMA Network Open*.

eTable 4: Clinical trial consents by intervention subgroup

	Control group	Intervention group	
Denominator	Trial consent rate (%, 95% CI)	Subgroup A trial consent rate (%, 95% CI)	Subgroup B trial consent rate (%, 95% CI)
Randomized patients (n=20707)	2.59 (2.24-2.99)	2.97 (2.60-3.40)	2.95 (2.58-3.38)
Randomized patients who had imaging studies (n=8277)	6.43 (5.57-7.41)	7.34 (6.42-8.38)	7.17 (6.27-8.19)
Randomized patients who had imaging studies and were ever "ready" per AI model (n=2127)	22.11 (19.18-25.35)	22.98 (20.02-26.23)	24.11 (21.15-27.34)
Randomized patients who started any new systemic therapy (n=2036)	23.31 (20.32-26.59)	27.59 (24.33-31.10)	27.45 (24.21-30.94)

Legend to eTable 4: Control group, control group with standard MatchMiner workflows; Subgroup A, intervention subgroup in which emails were automatically sent to treating oncologists when patients had genomic matches to a trial and were predicted likely to change treatment by our AI model; Subgroup B, intervention subgroup in which emails were sent to treating oncologists when patients had genomic matches to a trial, were predicted likely to change treatment, and did not meet exclusions on manual review. Manual review exclusions included uncontrolled brain metastases; poor performance status (ECOG performance status > 2); enrollment on hospice; multiple primary cancers; lack of measurable disease; AI model false positives; and already having received a new systemic therapy.

© 2025 Mazor T et al. *JAMA Network Open*.

eTable 5: Characteristics of physicians who received notifications

	N (%)
Total	151 (100)
Primary affiliation	
Academic disease center	
Breast	24 (16)
GI	21 (14)
GU	16 (11)
Thoracic	13 (9)
Non-oncology specialty	10 (7)
Other academic disease center	41 (27)
Regional affiliates	26 (17)
Sex	
Male	71 (47)
Female	80 (53)
Years in oncology practice*	
0-9	46 (30)
10-19	54 (36)
20-29	20 (13)
30+	11 (7)
Missing/unavailable	20 (13)

*Years in practice calculated as 2024 minus year of completion of clinical oncology training. Non-oncologists who received notifications (largely dermatologists in our cutaneous oncology center) were coded as missing/unavailable for years in practice.

© 2025 Mazor T et al. *JAMA Network Open*.

eFigure 1: Area under receiver operating characteristic curve of AI model for predicting new treatment

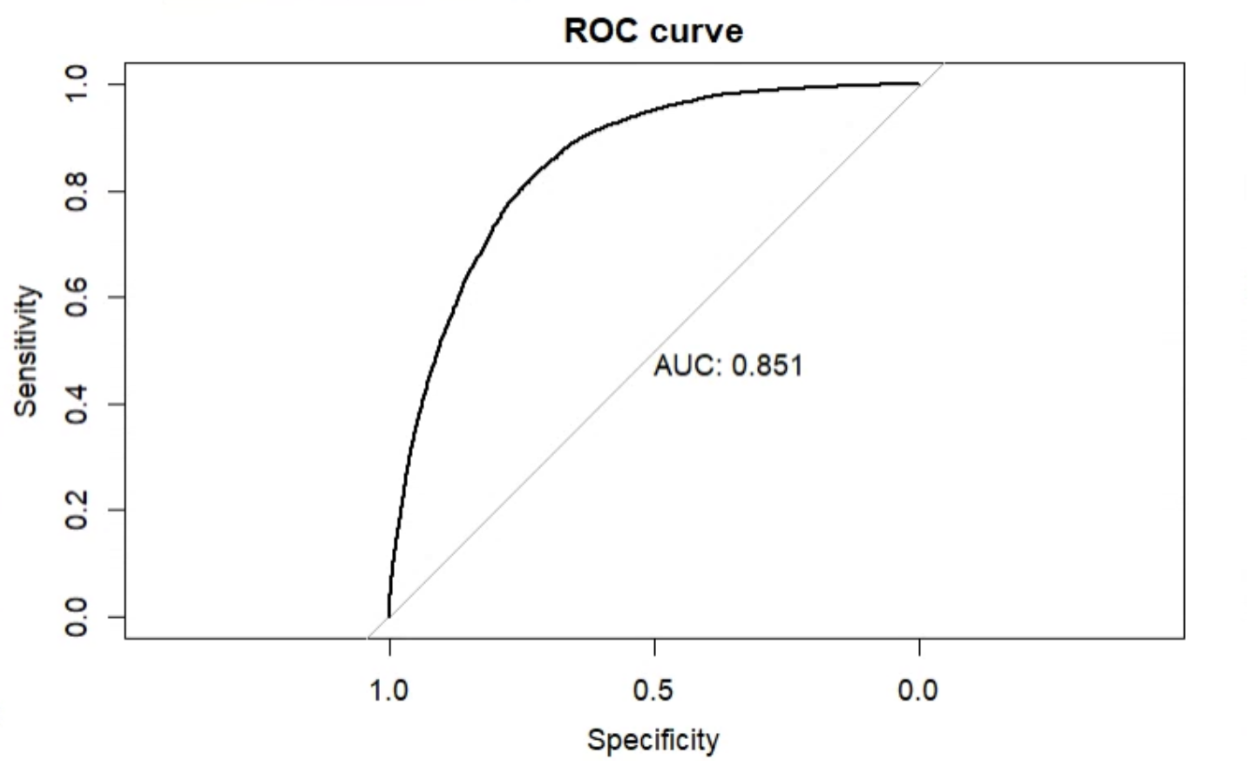

© 2025 Mazor T et al. *JAMA Network Open*.

eFigure 2: Area under precision recall curve for predicting new treatment

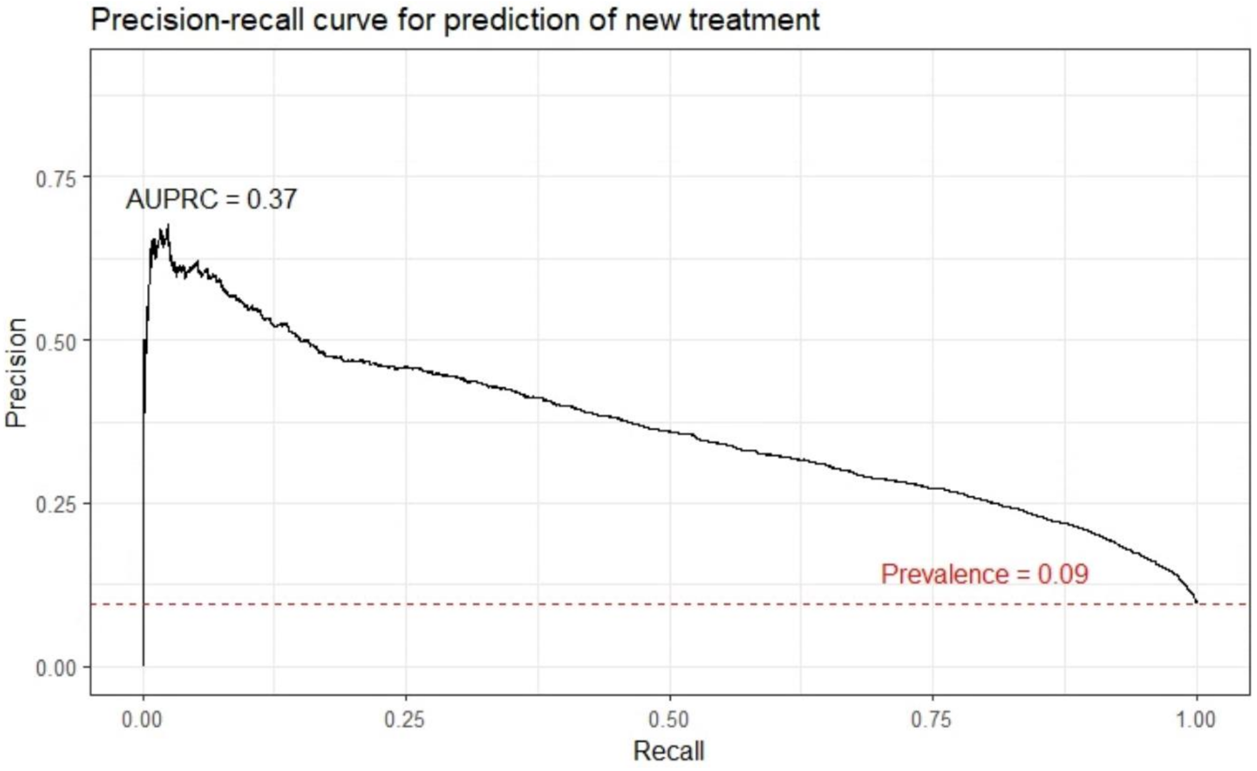

© 2025 Mazor T et al. *JAMA Network Open*.

eFigure 3: Confusion matrix for predicting new treatment at the best F_1 threshold

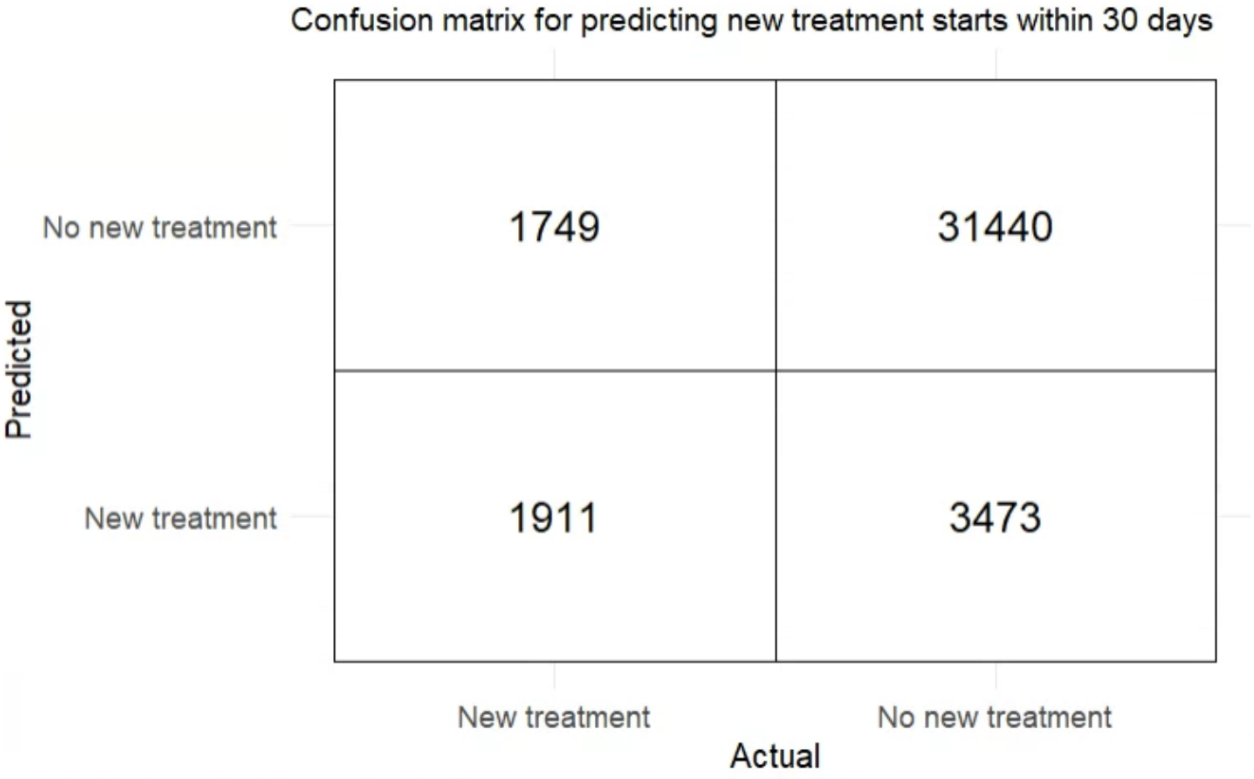

Statistics at the best F1 threshold:
Best F1 Score: 0.4226006
Best Threshold: 0.2589997

Accuracy : 0.8646, 95% CI : (0.8612, 0.868)
Kappa : 0.3491
Mcnemar's Test P-Value : <2e-16
Sensitivity : 0.52213
Specificity : 0.90052
Pos Pred Value : 0.35494
Neg Pred Value : 0.94730
Prevalence : 0.09489
Detection Rate : 0.04954
Detection Prevalence : 0.13958
Balanced Accuracy : 0.71133
'Positive' Class : New treatment

© 2025 Mazor T et al. *JAMA Network Open*.

eFigure 4: Distribution of new treatment predictions and calibration curve for predicting new treatment

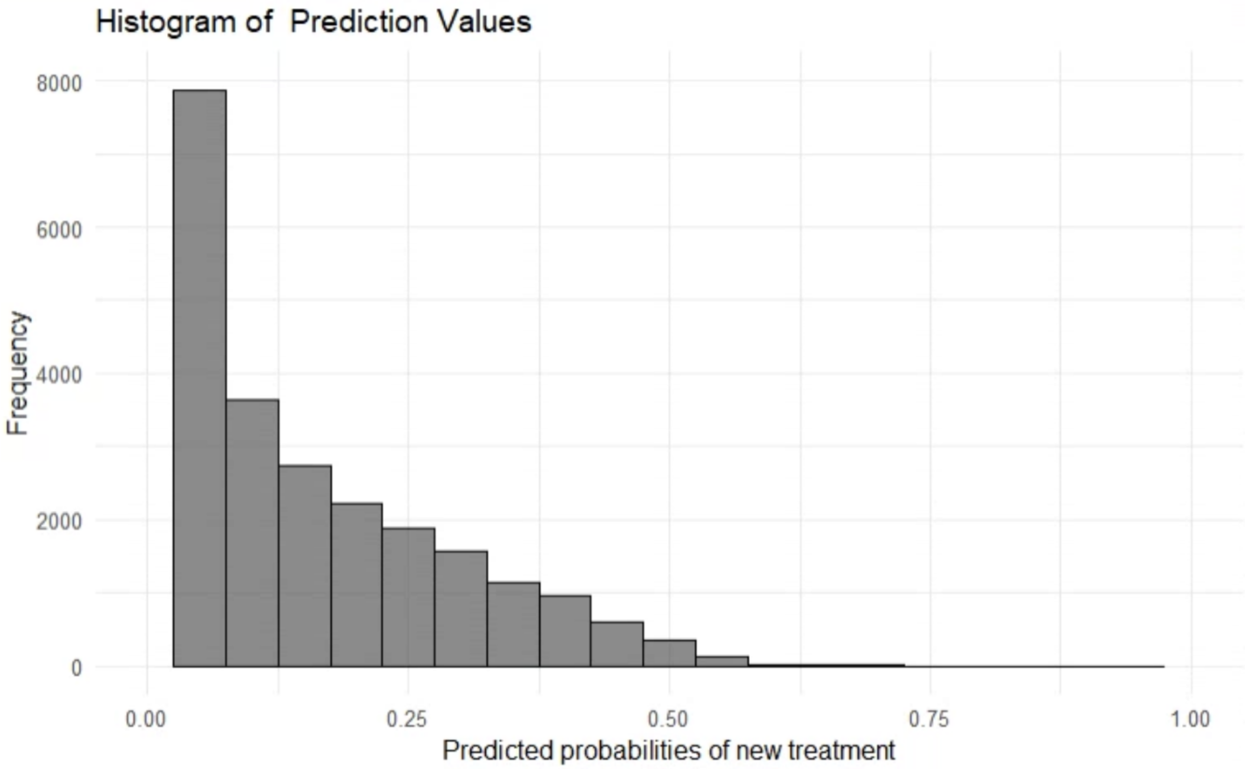

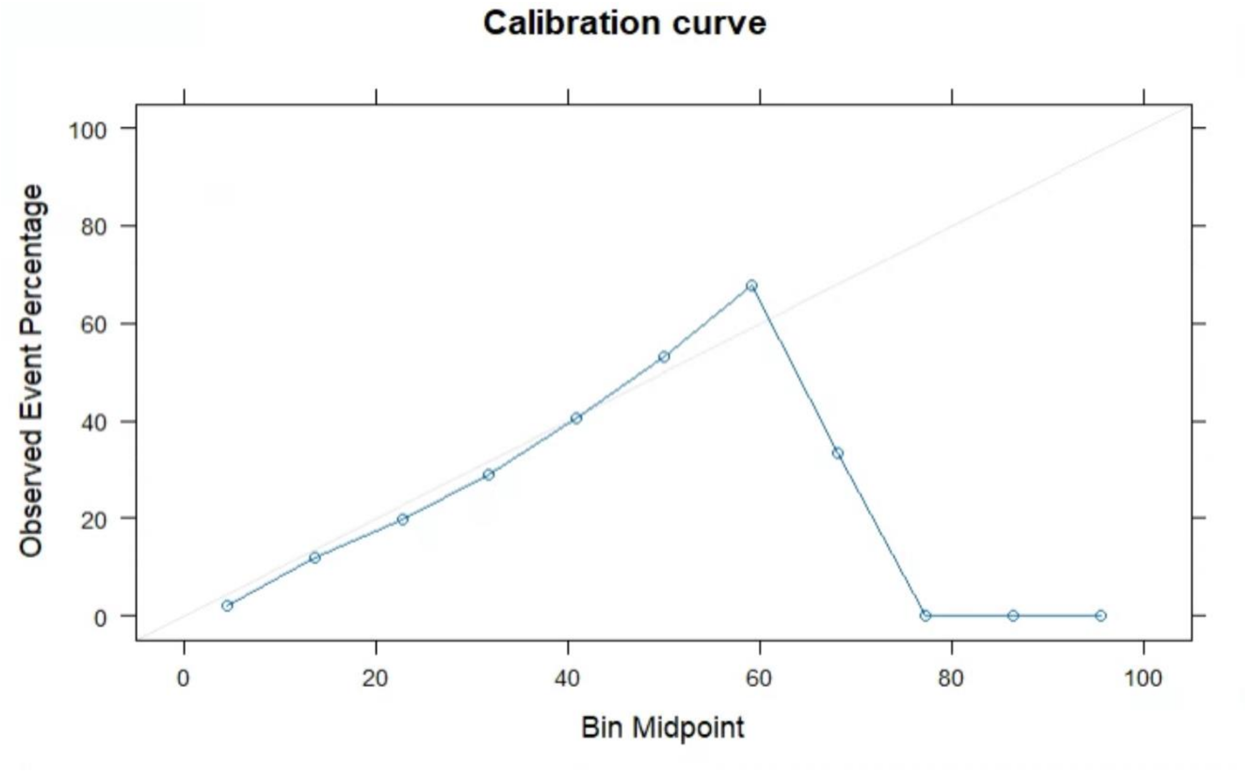

© 2025 Mazor T et al. *JAMA Network Open*.

eMethods

Physician assignment process

Each time the AI models flagged a patient, the patient was assigned to the physician with whom they had the most prior and upcoming appointments at Dana-Farber. Treating physicians were defined as follows. Providers with clinical encounters at Dana-Farber were identified. To enrich for physicians likely to make recommendations about genomically targeted clinical trials, clinicians were removed if they worked primarily in supportive care departments, such as psychiatry or palliative care. The physician list was manually reviewed, and physicians known to be surgeons were also removed. The main assignment process required that a patient have a minimum of two appointments with the top physician (looking back one year and including any future appointments scheduled for the next year). If the patient had the same number of appointments with two physicians, the patient was assigned to both physicians. These assignments were permanent for the duration of the intervention, and the same physician(s) would continue to receive a notification if the model flagged the patient again in the future. If no physician could be assigned with the main workflow, then a secondary workflow searched for any physician with whom the patient had a single appointment in the preceding three months or in the future. This was considered a temporary assignment and would not persist if the model flagged the patient again in the future.

© 2025 Mazor T et al. *JAMA Network Open*.

Example of email to treating oncologists (deidentified)

To Dr. Jones,

A DFCI machine learning model has identified that one of your patients has an elevated probability of changing treatment soon. We are sharing information about potential genomically-matched clinical trials for such patients as part of a study to evaluate the impact on trial accrual.

Patient MRN: 123456
Patient name: John Smith

We are collecting feedback on our model. Do you agree that your patient's disease status is such that considering new treatment is reasonable? Yes / No / Already changed treatment in past week

Current genomically driven trial matches from MatchMiner are listed below. Trial matches are based on the OncoPanel report from January 1, 2021.

A trial match does not guarantee trial eligibility; please refer to the protocol or contact the trial PI for complete eligibility requirements. Trials listed may not always have slots available.

Note that the trials listed below are matched to the patient based on genomic criteria only and are not a comprehensive list of treatment options. Please also consider standard of care therapies, non-genomically matched trials, and supportive care including palliative care consultation as clinically appropriate.

MatchMiner Trial Matches:

Genomic Match	Trial Information
**BRCA2 …**, Tier 2	IDE161 in Participants with Advanced Solid Tumors (23-600, Phase I)<br><br>Contact: DFCI/BWH Center for Cancer Therapeutic Innovation, Principal Investigator
**MTAP Homozygous deletion**	TNG462 in MTAP-Deleted Solid Tumors. (23-362, Phase I/II)<br><br>Contact: DFCI/BWH Center for Cancer Therapeutic Innovation, Principal Investigator

© 2025 Mazor T et al. *JAMA Network Open*.

**MTAP Homozygous deletion**	MRTX1719 in Advanced Solid Tumor with MTAP Deletion<br><br>(22-150, Phase I/II)<br><br>Contact: DFCI/BWH Thoracic Oncology, Principal Investigator
**MTAP Homozygous deletion**	IDE397 in Advanced Solid Tumors (21-552, Phase I)<br><br>Contact: DFCI/BWH Center for Cancer Therapeutic Innovation, Principal Investigator
**MTAP Homozygous deletion**	TNG908 in MTAP-Deleted Solid Tumors (22-280, Phase I/II)<br><br>Contact: MGH Thoracic Cancer, Principal Investigator

Trial matches are accurate as of the date of this email. Up-to-date trial matches can be found on the MatchMiner website or on the MatchMiner tab within Epic.

MatchMiner is only available within the Partners/MGB network or over VPN. Instructions to request access are available here.

To opt-out of future emails about this patient or all patients, or to have these emails sent to someone else, click here.

Additional Information:

OPTIONS (Optimizing Precision Trials with an artificial Intelligence driven Oncologist Notification System) is a study in which information about potential clinical trials derived from the MatchMiner engine is delivered to treating clinicians at moments in time when patients have an elevated predicted probability of starting new systemic therapy based on the text of their imaging reports. Predictions are derived from machine learning models trained using retrospective data to predict initiation of new treatment within 30 days and mortality within six months using the text of a given imaging report and the sequence of prior imaging reports for each patient (Kehl, K. et al.).

MatchMiner is a clinical trial matching system developed by the Knowledge Systems Group at DFCI. DFCI patients with OncoPanel sequencing data are matched nightly to genomically-driven clinical trials (Klein, H. et al.).

This email was automatically generated by MatchMiner on January 1, 2023.

© 2025 Mazor T et al. *JAMA Network Open*.
