# Supplementary material for: Clinical Trial Notifications Triggered by Artificial Intelligence–Detected Cancer Progression: A Randomized Trial
Source: JAMA Netw Open. 2025 Apr 21;8(4):e252013. doi: 10.1001/jamanetworkopen.2025.2013 (PMC12013351; doi:10.1001/jamanetworkopen.2025.2013)
Supplement: Supplement 3. — Data Sharing Statement [file jamanetwopen-e252013-s003.pdf]

Data Sharing Statement

Mazor. Clinical Trial Notifications Triggered by Artificial Intelligence–Detected Cancer Progression. *JAMA Netw Open*. Published April 21, 2025.
doi:10.1001/jamanetworkopen.2025.2013

Data
Additional Information: The current study was not itself considered a clinical trial by the NIH definition, since its endpoints were not health-related patient outcomes, and it was therefore not registered in clinicaltrials.gov.
Data available: No

Additional Information
Explanation for why data not available: The raw patient data used in this study include identified imaging reports, which cannot be made available due to patient privacy restrictions. On request, we can provide a deidentified version of the derived data used for our statistical analyses of associations between our intervention and process outcomes; this would consist of one row per patient and columns for intervention group, whether any imaging was performed, whether there were any AI model predictions of treatment change, whether any new treatment was started during the intervention, and whether the patient consented to and/or enrolled on a clinical trial during the intervention.
